# Age of Disease Onset and Risk of Serious Infection with anti-TNF Use in Older Adults with Rheumatoid Arthritis

**DOI:** 10.1093/geroni/igaf122.2413

**Published:** 2025-12-31

**Authors:** Jiha Lee, Sofia Pedro, Kaleb Michaud

**Affiliations:** University of Michigan, Ann Arbor, Michigan, United States; National Data Bank for Rheumatic Diseases, Wichita, Kansas, United States; University of Nebraska Medical Center, Omaha, Nebraska, United States

## Abstract

Older age increases the risk of serious infections (SI) in rheumatoid arthritis (RA) patients using biologics like anti-TNFs. About one-third of older adults are diagnosed with RA between ages 60-65, classified as late-onset RA (LORA). LORA exhibits more acute symptoms and faster disease progression compared to young-onset RA (YORA), possibly due to age-related immune changes. This study utilized data from the FORWARD-The National Databank for Rheumatic Diseases (2001-2019) to assess SI risk differences in older adults with LORA versus YORA starting anti-TNF therapy. Patients aged ≥60 years, categorized by age at RA diagnosis (LORA ≥60; YORA < 60), were propensity-matched for demographics and health factors. Exclusions applied to those with other rheumatic diseases, cancer, or HIV preceding SI. SI was defined as infections requiring hospitalization, IV antibiotics, or causing death, linked to anti-TNF within three months after treatment cessation. The study included 1,219 LORA and 6,030 weighted YORA patients. LORA patients had fewer previous infections, lower glucocorticoid (GC) use, but higher methotrexate use. Although LORA had a higher crude SI incidence (3.6/1000 patient-years) than YORA (2.7/1000 patient-years), SI risk did not significantly differ (aHR 0.85, 95% CI 0.58-1.25). Adjusting for prior SI, smoking, and methotrexate use did not alter outcomes. Long-term GC use and older age were independent risk factors for increased SI risk. These findings indicate that SI risk from anti-TNFs does not vary by RA onset age but highlight the risky and often suboptimal reliance on long-term GC use in LORA patients, suggesting a need for improved treatment strategies.

